# Marine and Semi-Synthetic Hydroxysteroids as New Scaffolds for Pregnane X Receptor Modulation

**DOI:** 10.3390/md12063091

**Published:** 2014-05-27

**Authors:** Valentina Sepe, Francesco Saverio Di Leva, Claudio D’Amore, Carmen Festa, Simona De Marino, Barbara Renga, Maria Valeria D’Auria, Ettore Novellino, Vittorio Limongelli, Lisette D’Souza, Mahesh Majik, Angela Zampella, Stefano Fiorucci

**Affiliations:** 1 Department of Pharmacy, University of Naples “Federico II”, Via D. Montesano, 49, I-80131 Napoli, Italy; E-Mails: valentina.sepe@unina.it (V.S.); fr.dileva@gmail.com (F.S.D.L.); carmen.festa@unina.it (C.F.); sidemari@unina.it (S.D.M.); madauria@unina.it (M.V.D.); ettore.novellino@unina.it (E.N.); vittoriolimongelli@gmail.com (V.L.); 2 Department Experimental and Clinical Medicine, University of Perugia, Via Gambuli 1, S. Andrea delle Fratte, Perugia 06132, Italy; E-Mails: claudiodamore1983@gmail.com (C.D.); barbara.renga@unipg.it (B.R.); stefano.fiorucci@unipg.it (S.F.); 3 CSIR-National Institute of Oceanography, Dona Paula, Goa 403004, India; E-Mails: lisette@nio.org (L.D.); mmajik@nio.org (M.M.)

**Keywords:** soft coral, *Sinularia kavarattiensis*, pregnane X receptor (PXR), hydroxysteroids, docking simulations

## Abstract

In recent years many sterols with unusual structures and promising biological profiles have been identified from marine sources. Here we report the isolation of a series of 24-alkylated-hydroxysteroids from the soft coral *Sinularia kavarattiensis*, acting as pregnane X receptor (PXR) modulators. Starting from this scaffold a number of derivatives were prepared and evaluated for their ability to activate the PXR by assessing transactivation and quantifying gene expression. Our study reveals that ergost-5-en-3β-ol (**4**) induces PXR transactivation in HepG2 cells and stimulates the expression of the PXR target gene CYP3A4. To shed light on the molecular basis of the interaction between these ligands and PXR, we investigated, through docking simulations, the binding mechanism of the most potent compound of the series, **4**, to the PXR. Our findings provide useful functional and structural information to guide further investigations and drug design.

## 1. Introduction

The pregnane X receptor (PXR, *NR1I2*) belongs to the nuclear receptor (NR) family and is well recognized for its pivotal role as a “xenobiotic sensor” that transcriptionally regulates the expression of Phase I and Phase II drug/xenobiotic metabolizing enzymes and transporters. The PXR has been detected in various tissues including kidney, colon, brain capillaries, small intestine, and predominantly in liver [1], and it can be activated by various ligands that can bind to the ligand binding domain (LBD). The pronounced flexibility of this ligand-binding pocket allows it to bind host molecules of different sizes and chemical structure. Thus, many prescription drugs, such as antibiotics, antineoplastic, anti-inflammatory and antihypertensive drugs [2] and several natural products [3,4] or herbal remedies have been reported to act as PXR agonists.

Activators of the PXR play a therapeutic role in the treatment of intestinal inflammation and of other immune-mediated dysfunctions in humans [5]. PXR agonists have been shown to attenuate inflammatory bowel disease by reducing nuclear factor-κB target gene expression that mediates colon inflammation [6,7,8,9,10].

Recent studies in our group led to the discovery of several molecules of marine origin with interesting profiles as NR modulators [3,11,12,13]. Among these, solomonsterols [14], truncated chain sulfated steroids, malaitasterol A [15], an unusual bis-secosterol, and gracilioethers [16] were endowed with selective activation action on the PXR, whereas theonellasterols and conicasterols showed a dual modulatory profile on the PXR and FXR [17,18,19,20,21]. The *in vivo* evaluation of a synthetic sample of solomonsterol A in a colitis model using transgenic mice expressing hPXR demonstrated the effectiveness of this PXR agonist in protecting the mouse against the development of the disease [7,8].

Pursuing our interest in the discovery of nuclear receptor (NR) modulators from marine sources, we analyzed the apolar extracts of the soft coral *Sinularia kavarattiensis*, collected in the Indian Ocean, that have afforded a family of conventional 3β-hydroxysteroids characterized by a differentiated pattern of alkylation on the side chain. Interestingly, transactivation assays on hPXR indicated that compound **4**, ergost-5-enol, was endowed with potent agonistic activity and its binding mechanism to PXR was elucidated through docking simulations. These findings prompted us to develop a library of simple mono hydroxylated steroidal derivatives, thus providing for the first time the structural basis of PXR modulation by conventional 3β-hydroxysteroid chemical scaffold.

## 2. Results and Discussion

### 2.1. Isolation of Cholesterol Derivatives with C-24 Alkylation from Indian Ocean Collection of Sinularia

**Figure 1:**
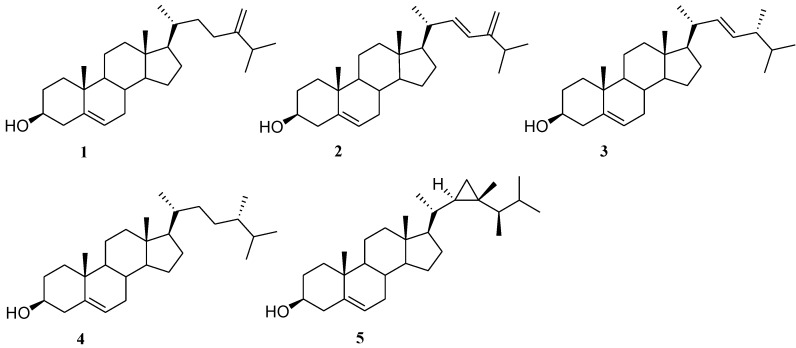
Cholesterol derivatives with C-24 alkylation from the soft coral *Sinularia* *kavarattiensis*.

The *n*-hexane extract (3.0 g) obtained by a solvent partitioning of the crude methanol extracts of *Sinularia kavarattiensis* was chromatographed by MPLC over silica gel, eluting with a gradient system of increasing polarity from dichloromethane to methanol, and the obtained fractions were further purified by analytical HPLC in MeOH/H_2_O (99:1) to afford 24-methylenecholesterol (**1**), ergosta-5,22,24(28)-trien-3β-ol (**2**), (24*R*)-ergosta-5,22-dien-3β-ol (**3**), (24*S*)-ergost-5-en-3β-ol (**4**) [22], and gorgosterol (**5**) [23]. The structures of the known compounds as reported in Figure 1 were assigned through comparison of their spectral data with those reported in the literature.

### 2.2. Preparation of 24-Methyl Stanols

Recently we demonstrated, that 4-methylenesteroids from *Theonella* sponges genus are endowed with peculiar pharmacological profiles on metabolic nuclear receptors, FXR and PXR [15]. All these molecules possess the unusual exocyclic double bound at C4 and the rare Δ^8,14^ on the tetracyclic nucleus and a 24-alkyl side chain with a 24*S*-ethyl group in the theonellasterol family or a 24*R*-methyl group in the conicasterol family. Indeed conicasterol, the ideal biomarker of *Theonella conica* [24], was proven to be a potent PXR agonist [14].

**Scheme 1:**
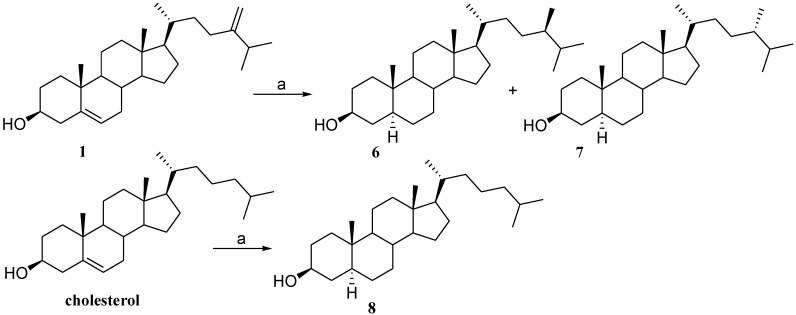
*Reagent and conditions*. (a) H_2_, PtO_2_, hexane dry (43% **6**, 57% **7**, 90% **8**).

Thus, we decided to explore the influence of the stereochemistry of the C-24 methyl group and of the rare 8,14 double bound on the activation of PXR. As depicted in Scheme 1, 24*R*- and 24*S*-methylcholestan-3β-ols (**6**,**7**) were obtained by hydrogenation of a sample of natural 24-methylenecholesterol (**1**) under platinum oxide (PtO_2_) catalyst. The obtained equimolar mixture of 24-methyl epimers was fractionated by reverse phase HPLC to afford pure 24*R*-methylcholestan-3β-ol (**6**) and 24*S*-methylcholestan-3β-ol (**7**).

**Scheme 2:**
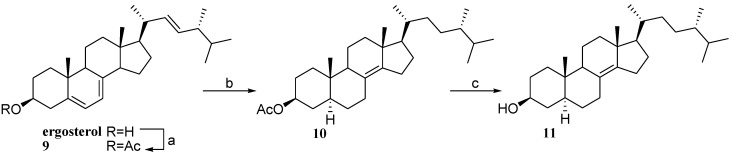
*Reagent and conditions*. (a) acetic anhydride, pyridine, quantitative yield; (b) H_2_, PtO_2_, AcOEt dry/AcOH 95:5 v/v, 95%; (c) *p*TsOH, CHCl_3_ dry/MeOH dry 5:3 v/v, 87%.

The C-24 configuration was assigned by comparison of ^1^H chemical shifts with those of epimeric steroidal side chain reported in the literature [25]. As a control sample in the evaluation of PXR modulation by 24-methyl sterol in transactivation assays, also cholestanol (**8**) was prepared through hydrogenation of a small sample of cholesterol (Scheme 1). Selective room temperature hydrogenation under platinum oxide catalyst of ergosterol acetate (**9**), afforded 3β-acetoxy-ergost-8(14)-ene (**10**) in 95% chemical yield [26]. As depicted in Scheme 2, removal of the acetyl protecting group led to ergost-8(14)-en-3β-ol (**11**), the Δ^8(14)^ derivative of 24*S*-stanol (**7**).

### 2.3. Preparation of Polar Side Chain Modified 3β-Hydroxy Steroids

**Scheme 3:**
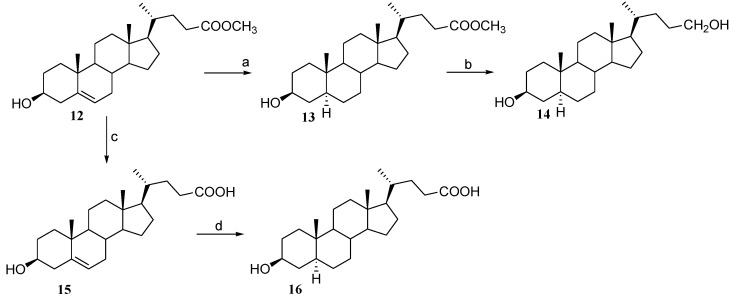
*Reagent and conditions*. (a) H_2_, Pd/C, THF dry/MeOH dry 1:1 v/v, quantitative yield; (b) LiBH_4_, MeOH dry, THF dry, 0 °C, 78%. (c) LiOH, THF/H_2_O 1:1 v/v, 80%; (d) H_2_, Pd/C, THF dry/MeOH dry 1:1 v/v, 93%.

As extensively demonstrated [27], PXR plays a key role in maintenance of bile acid (BAs) homeostasis. In fact, PXR is activated by the toxic bile acid lithocholic acid (LCA) and its 3-keto derivative thus functioning as physiological sensor of LCA and protecting the liver against severe damage induced by toxic bile acids [27]. Invariably BAs possess a carboxyl group at the C-24 position on their side chains and differ in the hydroxylation pattern of the A/B *cis* tetracyclic nucleus. Thus the introduction of a carboxy functional group on the side chain of tetracyclic nuclei with the A/B *trans* ring junction could be instrumental in the evaluation of PXR modulation by 3β,5α-hydroxy steroid scaffolds. Moreover, steroids with a polar group in the side chain should be conjugated with suitable carriers in the perspective to develop pro-drugs useful in tissue specific drug delivery [28]. First C-24 derivatives were prepared starting from methyl 3β-hydroxychol-5-en-24-oate (**12**) [18,29,30], whose Δ^5^ double bond was reduced affording the 5α-cholan methyl ester derivative **13** (Scheme 3).

LiBH_4_ treatment furnished the alcoholic function at C-24 in the derivative **14**. Methyl 3β-hydroxychol-5-en-24-oate (**12**) was also used as starting material to obtain carboxyl acid derivatives **15** and **16** through LiOH hydrolysis and then hydrogenation on palladium catalyst (Scheme 3).

**Scheme 4:**
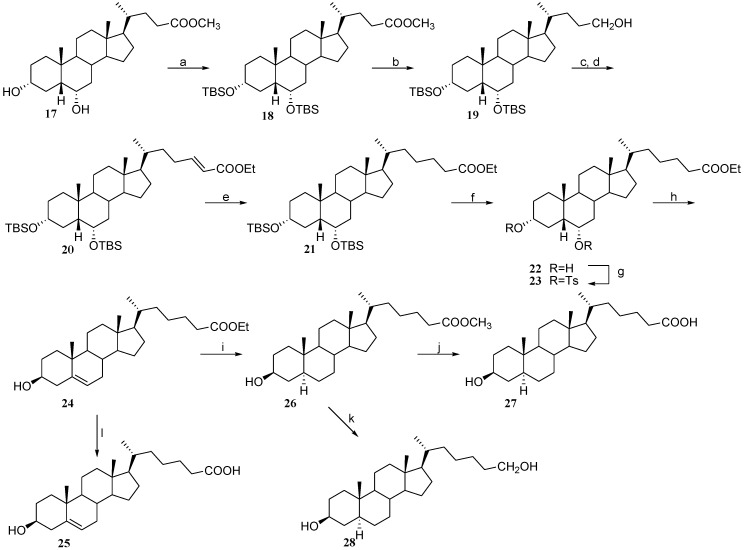
*Reagent and conditions*. (a) TBSOTf, lutidine, CH_2_Cl_2_ dry, quantitative yield; (b) LiBH_4_, MeOH dry, THF dry, 0 °C, 93%; (c) oxalyl chloride, DMSO, TEA dry, CH_2_Cl_2_ dry, −78 °C; (d) LiOH, triethyl phosphonoacetate, THF dry, reflux, 95% over two steps; (e) H_2_, Pd/C, THF dry/MeOH dry 1:1 v/v, 98%; (f) HCl 37%, EtOH dry; (g) TsCl, pyridine dry, 90% over two steps; (h) AcOK, DMF/H_2_O 7:1 v/v, reflux then *p*TsOH, CHCl_3_ dry/MeOH dry 5:3 v/v, 78% over two steps; (i) H_2_, Pd/C, THF dry/MeOH dry 1:1 v/v, 91%; (j) NaOH 10% in MeOH/H_2_O 2:1 v/v, quantitative yield; (k) LiBH_4_, MeOH dry, THF dry, 0 °C, 78%; (l) LiOH, THF/H_2_O 1:1 v/v, quantitative yield.

The synthesis of C-26 3β-hydroxy steroids started from commercially available hyodeoxycholate (**17**) which was protected at C-3 and C-6 to give the corresponding 3,6-disilyl derivative **18** (Scheme 4). LiBH_4_ reduction of the C-24 ester function in dry methanol afforded the C-24 primary alcohol (**19**) in nearly quantitative yield. One pot Swern oxidation to aldehyde followed by Horner C-2 homologation led to *trans*-α,β-unsaturated ester (**20**) that was hydrogenated to the corresponding saturated ester (**21**) in 98% yield. Removal of the silyl protective groups and further tosylation with tosyl chloride in pyridine afforded the C-26 3,6-ditosylate derivative **23** used as key intermediate for the synthesis of C-26 polar side chain derivatives. As previously reported [25], strong base treatment of the tosylated derivative proceeded with simultaneous elimination at C-6 and to inversion at C-3 to give ethyl 3β-hydroxy-5-cholen-26-oate (**24**). The corresponding carboxy acid derivative (**25**) was obtained by alkaline hydrolysis of **24** with LiOH in THF/H_2_O 1:1. Hydrogenation of the Δ^5^ double bond allowed installation of the desired *trans* A/B junction and proceeded with concomitant *trans-*esterification induced by methanolic solvent. The so obtained methyl ester (**26**) was hydrolyzed to the carboxy derivative (**27**), or, alternatively reduced to the primary alcohol (**28**) by treatment with LiBH_4_.

### 2.4. Pharmacological Evaluation

The natural derivatives **1**–**5**, the 24-methyl stanols (**6**–**8**, **11**) and the 3β-hydroxy steroids with polar side chains (**12**–**16** and **24**–**28**) were evaluated as PXR modulators in a luciferase reporter assay, in the presence and absence of rifaximin (10 μM), a well characterized PXR agonist [31] on a human hepatocyte cell line (HepG2 cells) transiently transfected with pSG5-PXR, pSG5-RXR, pCMV-β galactosidase, and p(CYP3A4)-TK-Luc vectors.

**Figure 2:**
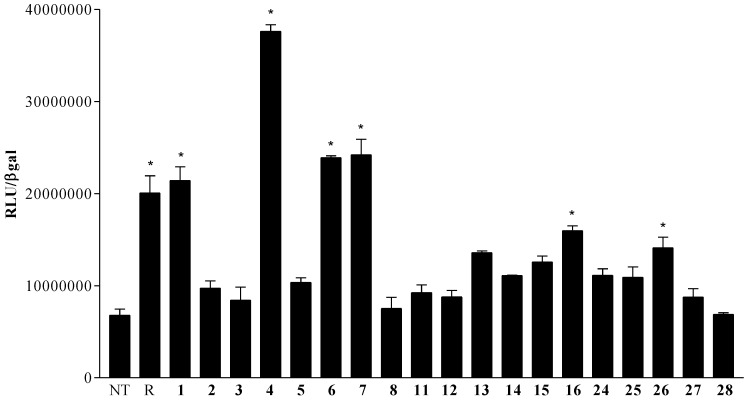
Pregnane X receptor (PXR)transactivation assay in HepG2 cells; 24 h post transfection with pSG5-PXR, pSG5-RXR, pCMV-βgal, and p(CYP3A4)TKLUC vectors, HepG2 cells were incubated with rifaximin (R) 10 μM or compounds **1**–**8**, **11**–**16** and **24**–**28** 10 μM for 18 h. * *p* < 0.05 *vs.* not treated (NT).

Data shown in Figure 2 are quite interesting. As expected, cholestanol (**8**) was not able to transactivate PXR at 10 μM, when administrated alone. At variance with **8**, both derivatives **1** and **4**, obtained through the substitution at C-24 on the side chain of a Δ^5^ cholesten nucleus with an exomethylene functionality and a (*S*)-methyl group, respectively, show PXR agonistic activity with compound **4** the most potent activator identified in this study.

On the contrary, the introduction of an additional unsaturation on the side chain (Δ^22^ in **2** and **3**) or a cyclopropane ring as in **5** caused a dramatic loss in the biological activity, thus suggesting a relevant role of the ligand side chain during the binding to the PXR-LBD. Of interest, regardless of the stereochemistry at C-24, the 24-methyl cholestanol derivatives, **6** and **7**, transactivated the PXR with a potency comparable to rifaximin. Comparing the different activity of derivative **11** (Scheme 2) and **7** (Scheme 1) and looking at their chemical structures, it can be observed that the introduction of a double bond in ring C, as in the case of **11**, causes a drastic decrease of the agonistic activity, that can be explained by the different conformation assumed by the tetracyclic nucleus. Even steroids with different polar side chains (**12**–**16** and **24**–**28** in Figure 2) were almost inactive with the exception of the C-24 carboxyl acid derivative, **16**, and the C-26 methyl ester derivative, **26**, that retain a slight agonism towards PXR.

**Figure 3:**
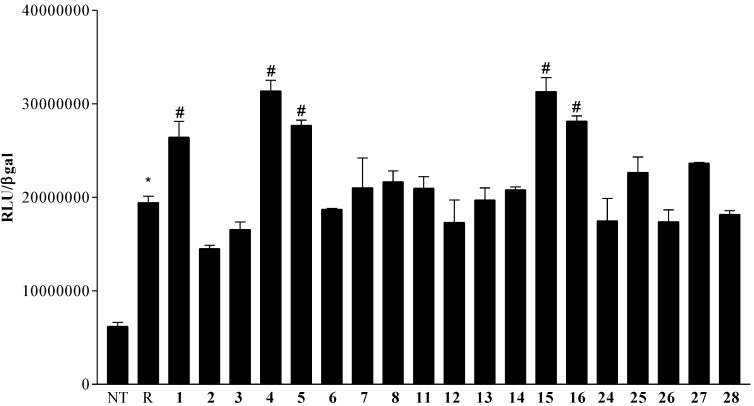
PXR transactivation assay in HepG2 cells; 24 h post transfection with pSG5-PXR, pSG5-RXR, pCMV-β-galactosidase, and p(CYP3A4)TKLUC vectors, HepG2 cells were incubated with rifaximin (R) 10 μM in combination with compounds **1**–**8**, **11**–**16** and **24**–**28** 50 μM for 18 h. * *p* < 0.05 *vs.* not treated (NT); # *p* < 0.05 *vs.* R.

Data from cell stimulation in presence of rifaximin (Figure 3) reveal that none of the tested compounds was relatively effective in inhibiting PXR transactivation caused by rifaximin, thus none of them showed an antagonistic profile.

#### Pharmacologial evaluation on **4**

**Figure 4:**
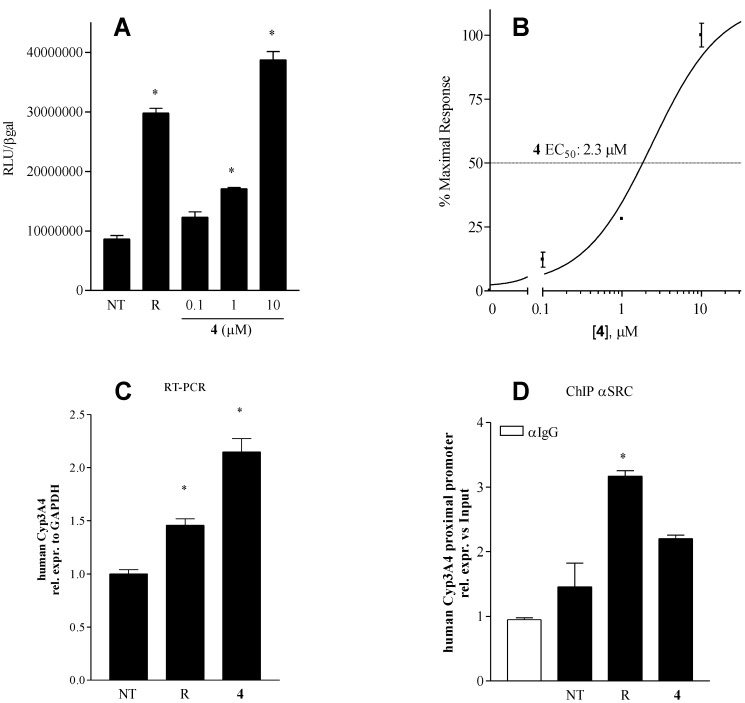
(**A**,**B**) Dose-response curve; HepG2 cells, transfected for PXR transactivation assay as described above, were stimulated with increasing concentration of compound **4** (0.1, 1 and 10 μM). Data obtained from transactivation experiments (**A**) were used for determination of compound 4 EC_50_ value (**B**), * *p* < 0.05 *vs.* not treated (NT); (**C**) Real-Time PCR analysis of CYP3A4 gene expression. HepG2 cells were treated for 18 h with rifaximin (R) 10 μM or with compound **4** 10 μM, * *p* < 0.05 *vs.* not treated (NT); (**D**) Chromatin immunoprecipitation assay carried out to detect the interaction of PXR, SRC-1 with the CYP3A4 promoter.

A concentration-response curve was then obtained for the most potent derivative **4**. As shown in Figure 4, Panels A and B, we found that this compound transactivates the PXR with an EC_50_ of ~2 μM with an efficacy of 140% with respect to rifaximin, thus confirming that this compound is a potent PXR agonist. To give support to the agonism of **4**, we then tested its effect on the expression of CYP3A4 that is targeted by rifaximin in a PXR dependent manner. Results shown in Figure 4, Panels C, demonstrate that compound **4** is a potent inductor of the expression of CYP3A4, a canonical PXR target gene, thus confirming **4** as a PXR agonist.

To gain further insights into the molecular mechanism mediating the agonistic activity of **4**, we then investigated the effect of this agent on the recruitment of SRC-1, a well characterized PXR co-activator [32], in chromatin immunoprecipitation (ChIP) experiments. As shown in Figure 4, Panel D, we found that exposure of HepG2 cells to rifaximin induces the recruitment of SRC-1 to a PXR responsive element in the CYP3A4 promoter. Of relevance, a similar positive interaction was detected in cells exposed to compound **4** (Figure 4D).

### 2.5. Binding Mode of Compound 4

**Figure 5:**
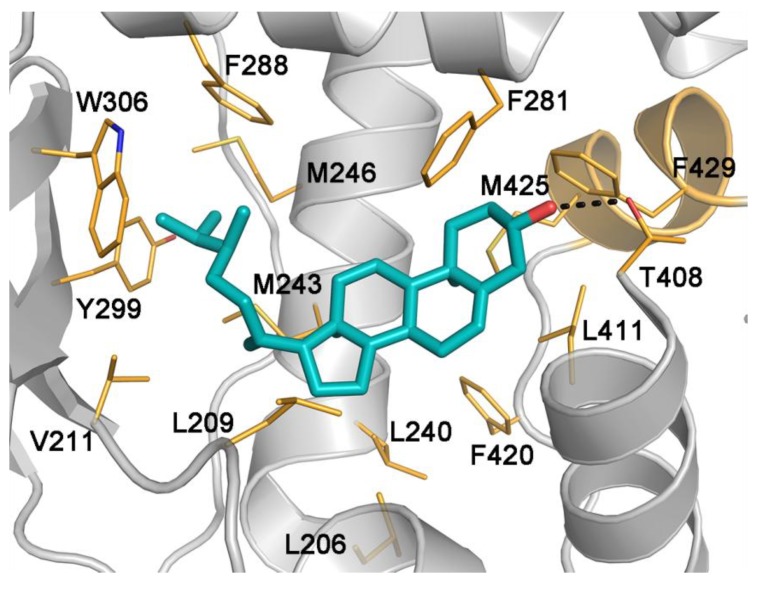
Binding mode of the PXR agonist **4** (cyan sticks) in the PXR-LBD crystal structure (PBD code 3hvl). PXR is shown as gray cartoon, while the AF-2 helix is colored in orange. Amino acids involved in ligand binding are shown as orange sticks. Residues from Pro268 to Arg287, from Gln316 to His336 and Asn380 to His407, and all hydrogens are omitted for clarity.

Prompted by the promising pharmacological data, we decided to elucidate the binding mode of compound **4**, the most potent derivative of the series, through docking simulations. For these calculations, we used the crystal structure of the PXR-LBD in complex with the inhibitor SR-12813 (PDB code 3hvl), which has been successfully employed to investigate the binding of ligands with steroidal scaffold to PXR [33,34]. In the best-scored docking pose, the ligand occupies the binding site pointing its polar head towards the activation function-2 domain (AF-2, colored in orange in Figure 5). Here, the hydroxyl group on the ring A of **4** is involved in H-bonds with the hydroxyl group of Thr408, while the steroidal scaffold engages hydrophobic contacts with residues such as Leu240, Met243, Phe281, Leu411, Phe420, Met425 and Phe429. It is worth noting that Met425 and Phe429 are on a small helix of the AF-2 domain. This domain undergoes large conformational changes upon ligand binding, changing the receptor binding affinity for co-activator and co-repressor peptides and thus regulating the transcription of target genes [35,36]. In the present case, **4** engages a network of lipophilic interactions involving residues Met425 and Phe429 on the AF-2 helix in the PXR-LBD. This hydrophobic cluster stabilizes the receptor conformation competent for the recruitment of co-activator peptides, thus enhancing the transcription of target genes. 

A similar functional mechanism was very recently reported by us and other authors for different nuclear receptors [37,38,39,40,41]. On the other side of the binding site, the flexible tail of **4** deepens into a narrow pocket where it establishes a series of hydrophobic contacts with residues such as Leu206, Leu209, Val211, Met243, Met246, Phe288, Trp299 and Tyr306. These favorable interactions further stabilize the ligand binding mode.

## 3. Experimental Section

### 3.1. Chemistry

#### 3.1.1. General Procedures

Specific rotations were measured on a Jasco P-2000 polarimeter (Jasco, Inc. Easton, MD, USA). High-resolution ESI-MS spectra were performed with a Micromass Q-TOF mass spectrometer (Waters Corporation, Milford, MA, USA). NMR spectra were obtained on Varian Inova 400 and Varian Inova 700 NMR spectrometers (Varian Medical System, Inc., Palo Alto, CA, USA) (^1^H at 400 and 700 MHz, ^13^C at 100 MHz) equipped with a Sun hardware and recorded in CDCl_3_ (δ_H_ = 7.26 and δ_C_ = 77.0 ppm), CD_3_OD (δ_H_ = 3.30 and δ_C_ = 49.0 ppm) and C_6_D_6_ (δ_H_ = 7.16 and δ_C_ = 128.4 ppm). *J* are in hertz and chemical shifts (δ) are reported in ppm and referred to CHCl_3_, CHD_2_OD and C_6_HD_5_ as internal standards. HPLC was performed using a Waters Model 510 pump (Waters corporation, Milford, MA, USA) equipped with Waters Rheodine injector (Waters corporation, Milford, MA, USA) and a differential refractometer, model 401 (Waters corporation, Milford, MA, USA). Reaction progress was monitored via thin-layer chromatography (TLC) on Alugram^®^ silica gel G/UV254 plates (Macherey-Nagel, GmbH & Co. KG, Düren, Germany). Silica gel MN Kieselgel 60 (70–230 mesh) from Macherey-Nagel Company, Düren, Germany was used for column chromatography. All chemicals were obtained from Sigma-Aldrich, Inc., St. Louis, MO, USA. Solvents and reagents were used as supplied from commercial sources with the following exceptions. Hexane, ethyl acetate, chloroform, dichloromethane, tetrahydrofuran and triethylamine were distilled from calcium hydride immediately prior to use. Methanol was dried from magnesium methoxide as follow. Magnesium turnings (5 g) and iodine (0.5 g) are refluxed in a small (50–100 mL) quantity of methanol until all of the magnesium has reacted. The mixture is diluted (up to 1 L) with reagent grade methanol, refluxed for 2–3 h then distilled under nitrogen. All reactions were carried out under argon atmosphere using flame-dried glassware. 

The purity of all of the intermediates, checked by ^1^H NMR, was greater than 95%.

#### 3.1.2. Isolation Procedures

*Sinularia kavarattiensis* Alderslade & Prita, collected off the coast of Rameshwaram, Tamil Nadu, India (Latitude: 9°16′60″ N Longitude: 79°17′60″ E) in December 2010, was frozen at −20 °C and transferred to the Council of Scientific and Industrial Research-National Institute of Oceanography (CSIR-NIO) Laboratory, Goa, India. The organism was identified by Dr. P. A. Thomas, Emeritus Scientist, Vizhingam Research Center, Central Marine Fisheries Research Institute, Kerala, India. A voucher specimen (14S021) is deposited at the CSIR-NIO. 

Freeze-dried organism (400 g) was extracted with 80% methanol (500 mL × 4) to obtain 23 g of the crude methanolic extract that was subjected to a modified Kupchan’s partitioning procedure as follows. The methanol extract was dissolved in a mixture of MeOH/H_2_O containing 10% H_2_O and partitioned against *n*-hexane to give 2.9 g of the crude extract. The water content (% v/v) of the MeOH extract was adjusted to 30% and partitioned against CHCl_3_ to give 3.2 g of the crude extract The aqueous phase was concentrated to remove MeOH and then extracted with *n*-BuOH (1.6 g of crude extract). The *n*-hexane extract (2.9 g) was fractionated by silica gel MPLC using a solvent gradient system from CH_2_Cl_2_ to MeOH. The fractions eluted with CH_2_Cl_2_/MeOH 995:5 and 992:8 (198 mg) were further purified by HPLC on a Nucleodur 100-5 C18 (5 μm; 4.6 mm i.d. × 250 mm, Macherey-Nagel, GmbH & Co. KG, Düren, Germany) with MeOH/H_2_O 99:1 as eluent (flow rate 1 mL/min) to give 1.3 mg of ergosta-5,22,24(28)-trienol (**2**) (*t*_R_ = 16 min), 6.9 mg of 24-methylenecholesterol (**1**) (*t*_R_ = 24 min), 2.6 mg of (24*R*)-ergosta-5,22-dienol (**3**) (*t*_R_ = 26 min), 5.3 mg of (24*S*)-ergost-5-enol (**4**) (*t*_R_ = 32 min) and 4.2 mg of gorgosterol (**5**) (*t*_R_ = 42.5 min).

**24-methylenecholesterol (1)**: white amorphous solid; 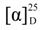 −16.6 (*c* 0.04, CHCl_3_); HRMS-ESI *m*/*z* 399.3625 [M + H]^+^, C_28_H_47_O requires 399.3627. Selected ^1^H NMR (C_6_D_6_): δ_H_ 5.36 (br d, *J* = 5.0 Hz, 1H), 4.92 (s, 1H), 4.90 (s, 1H), 3.40 (m, 1H), 1.09 (d, *J* = 6.8 Hz, 3H), 1.07 (d, *J* = 6.8 Hz, 3H), 1.01 (d, *J* = 6.8 Hz, 3H), 0.95 (s, 3H), 0.66 (s, 3H).

**Ergosta-5,22,24(28)-trien-3β-ol (2)**: white amorphous solid; 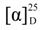 −38.7 (*c* 0.05, CHCl_3_); HRMS-ESI *m*/*z* 397.3464 [M + H]^+^, C_28_H_45_O requires 397.3470. Selected ^1^H NMR (C_6_D_6_): δ_H_ 6.07 (d, *J* = 15.6 Hz, 1H), 5.63 (dd, *J* = 8.7, 15.6 Hz, 1H), 5.36 (br d, *J* = 3.9 Hz, 1H), 5.03 (s, 1H), 4.96 (s, 1H), 3.40 (m, 1H), 1.14 (d, *J* = 7.0 Hz, 6H), 1.10 (d, *J* = 7.0 Hz, 3H), 0.95 (s, 3H), 0.66 (s, 3H).

**(24*R*)-ergosta-5,22-dien-3β-ol (3)**: white amorphous solid; 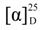 −23.6 (*c* 0.04, CHCl_3_); HRMS-ESI *m*/*z* 399.3622 [M + H^+^], C_28_H_47_O requires 399.3627. Selected ^1^H NMR (C_6_D_6_): δ_H_ 5.36 (br d, *J* = 5.0 Hz, 1H), 5.29 (ovl, 1H), 5.27 (ovl, 1H), 3.39 (m, 1H), 1.12 (d, *J* = 7.0 Hz, 3H), 1.01 (d, *J* = 7.0 Hz, 3H), 0.95 (s, 3H), 0.92 (d, *J* = 6.8 Hz, 6H), 0.68 (s, 3H).

**(24*S*)-ergost-5-en-3β-ol (4)**: white amorphous solid; 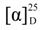 −42.5 (*c* 0.04, CHCl_3_); HRMS-ESI *m*/*z* 401.3778 [M + H]^+^, C_28_H_49_O requires 401.3783. NMR data as previously reported [19].

**Gorgosterol (5)**: white amorphous solid; 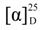 −29.5 (*c* 0.06, CHCl_3_); HRMS-ESI *m*/*z* 443.4249 [M + H]^+^, C_31_H_55_O requires 443.4253. NMR data as previously reported [20].

#### 3.1.3. Synthesis

**24*R*-methylcholestan-3β-ol and 24*S*-methylcholestan-3β-ol (6, 7)**. A solution of ergosta-5,22,24(28)-trienol (**1**) (5 mg, 0.01 mmol) in hexane dry (5 mL) was hydrogenated in the presence of platinum (IV) oxide (2 mg). The flask was evacuated and flushed first with argon and then with hydrogen. The reaction was stirred at room temperature under H_2_ for 5 min. The catalyst was filtered through silice, and the filtrate was concentrated under vacuum. The mixture was purified by HPLC on a Nucleodur Isis 100-5 C18 (5 μm; 4.5 mm i.d. × 250 mm, Macherey-Nagel, GmbH & Co. KG, Düren, Germany) with MeOH/H_2_O (999.5:0.5) as eluent (flow rate 1 mL/min) to give 1.8 mg (43% from **1**) of **6** (*t*_R_ = 27 min) and 2.4 mg (57% from **1**) of **7** (*t*_R_ = 28 min) as amorphous solids.

**24*R*-methylcholestan-3β-ol (6)**. 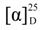 +0.4 (*c* 0.09, CH_3_OH); selected ^1^H NMR (700 MHz, C_6_D_6_): δ_H_ 3.37 (m, 1H), 2.99 (d, *J* = 5.0 Hz, 1H), 1.02 (d, *J* = 6.8 Hz, 3H), 0.93 (d, *J* = 6.8 Hz, 3H), 0.91 (d, *J* = 6.8 Hz, 3H), 0.87 (d, *J* = 6.8 Hz, 3H), 0.70 (s, 3H), 0.66 (s, 3H). HRMS-ESI *m*/*z* 403.3937 [M + H]^+^, C_28_H_51_O requires 403.3940.

**24*S*-methylcholestan-3β-ol (7)**. 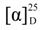 +0.6 (*c* 0.12, CH_3_OH); selected ^1^H NMR (700 MHz, C_6_D_6_): δ_H_ 3.40 (m, 1H), 3.01 (d, *J* = 5.0 Hz, 1H), 1.02 (d, *J* = 6.8 Hz, 3H), 0.92 (d, *J* = 6.8 Hz, 3H), 0.87 (d, *J* = 6.8 Hz, 3H), 0.85 (d, *J* = 6.8 Hz, 3H), 0.70 (s, 3H), 0.66 (s, 3H). HRMS-ESI *m*/*z* 403.3935 [M + H]^+^, C_28_H_51_O requires 403.3940.

**Cholestanol (8)**. The same reaction was carried out on cholesterol (10 mg, 0.025 mmol) to give cholestanol **8** (8.7 mg, 0.022 mmol, 90%). 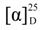 +11.8 (*c* 0.27, CHCl_3_); selected ^1^H NMR (400 MHz, CDCl_3_): δ_H_ 3.58 (m, 1H), 0.89 (d, *J* = 6.0 Hz, 3H), 0.85 (d, *J* = 6.0 Hz, 6H), 0.79 (s, 3H), 0.64 (s, 3H). ^13^C NMR (100 MHz, CDCl_3_): δ_C_ 71.7, 56.8, 56.5, 54.6, 45.1, 42.8, 40.3, 39.8, 38.5, 37.2, 36.4, 36.1, 35.8, 35.7, 32.4, 31.8, 29.0, 28.5, 28.2, 24.5, 24.0, 23.0, 22.8, 21.5, 18.9, 12.6, 12.3; HRMS-ESI *m*/*z* 389.3781 [M + H]^+^, C_27_H_49_O requires 389.3783.

**3β-*O*-acetyl-ergosterol (9)**. To a solution of ergosterol (100 mg, 0.25 mmol) in dry pyridine (5 mL) was added acetic anhydride (250 μL, 2.5 mmol). The mixture was left to stand at room temperature for 3 h. Then the solvent was evaporated. Purification by silica gel eluting with *n*-hexane/ethyl acetate 995:5 gave the acetyl ester **9** as an amorphous solid (108 mg, quantitative yield). An analytic sample was obtained by silica gel chromatography eluting with CH_2_Cl_2_. 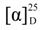 −86.0 (*c* 0.07, CHCl_3_); selected ^1^H NMR (400 MHz, CDCl_3_): δ_H_ 5.56 (m, 1H), 5.37 (m, 1H), 4.70 (m, 1H), 2.50 (m, 1H), 2.36 (m, 1H), 2.04 (s, 3H), 1.03 (d, *J* = 6.8 Hz, 3H), 0.95 (s, 3H), 0.91 (d, *J* = 6.8 Hz, 3H), 0.84 (d, *J* = 6.4 Hz, 3H), 0.82 (d, *J* = 6.4 Hz, 3H), 0.62 (s, 3H). ^13^C NMR (100 MHz, CDCl_3_): δ_C_ 170.5, 141.7, 138.8, 135.6, 132.0, 120.2, 116.2, 72.7, 55.7, 54.5, 46.0, 42.7 (2C), 40.4, 38.9, 37.8, 37.0, 36.6, 33.0, 28.2, 28.0, 22.9, 21.3, 21.0, 20.9, 19.8, 19.6, 17.5, 16.0, 12.0; HRMS-ESI *m*/*z* 439.6920 [M + H]^+^, C_30_H_47_O_2_ requires 439.6924.

**3β-acetoxy-ergost-8(14)-ene (10)**. Compound **9** (100 mg, 0.23 mmol) in a mixture of ethyl acetate and glacial acetic acid 95:5 v/v (10 mL) was hydrogenated under H_2_ in Parr apparatus at 3 atm for 48 h in the presence of a PtO_2_ catalyst (10 mg).The mixture was filtered through Celite, and the recovered filtrate was concentrated to give 96.5 mg of pure **10** as an amorphous solid (95%). An analytic sample was obtained by silica gel chromatography eluting with *n*-hexane/ethyl acetate 99:1.


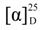
 +1.23 (*c* 0.19, CHCl_3_); selected ^1^H NMR (400 MHz, CDCl_3_): δ_H_ 4.71 (m, 1H), 2.02 (s, 3H), 0.92 (d, *J* = 6.7 Hz, 3H), 0.84 (d, *J* = 6.7 Hz, 3H), 0.83 (s, 3H), 0.77 (d, *J* = 6.5 Hz, 6H), 0.69 (s, 3H). ^13^C NMR (100 MHz, CDCl_3_): δ_C_ 170.7, 143.0, 126.2, 73.7, 56.7, 49.2, 44.1, 42.7, 39.1, 37.3, 36.7, 36.3, 34.8, 34.1, 33.5, 31.5, 30.4, 29.5, 28.7, 27.5, 27.0, 25.8, 21.5, 20.5, 19.9, 19.3, 18.2, 17.6, 15.4, 12.7. HRMS-ESI *m*/*z* 443.3885 [M + H]^+^, C_30_H_51_O_2_ requires 443.3889.

**5α-ergost-8(14)-en-3β-ol (11)**. Compound **10** (95 mg, 0.21 mmol) was dissolved in 8 mL of mixture CHCl_3_ dry/MeOH dry (5:3). Then to a solution was added *p*-toluensulfonic acid (*p*TsOH) (190 mg, 1 mmol). The mixture was quenched by addition of NaHCO_3_ solution (30 mL) and then concentrated *in vacuo*. Ethyl acetate and water were added and the separated aqueous phase was extracted with ethyl acetate (3 × 50 mL). The combined organic phases were washed with water, dried (Na_2_SO_4_) and concentrated. Purification by silica gel eluting with *n*-hexane/ethyl acetate (8:2) gave the alcohol **11** as a white solid (73 mg, 87%). 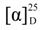 +10.2 (*c* 0.13, CHCl_3_); selected ^1^H NMR (400 MHz, CDCl_3_): δ_H_ 3.62 (m, 1H), 0.93 (d, *J* = 6.5 Hz, 3H), 0.85 (d, *J* = 6.4 Hz, 3H), 0.84 (s, 3H), 0.78 (d, *J* = 6.5 Hz, 6H), 0.63 (s, 3H); ^13^C NMR (100 MHz, CDCl_3_): δ_C_ 142.6, 126.3, 71.2, 56.6, 49.2, 44.2, 42.7, 39.0, 38.2, 37.2, 36.7, 36.5, 34.8, 33.5, 31.5, 30.3, 29.6, 28.8, 27.0 (2C), 25.8, 20.5, 19.9, 19.2, 18.2, 17.5, 15.4, 12.8; HRMS-ESI *m*/*z* 401.3780 [M + H]^+^, C_28_H_49_O requires 401.3783.

**Methyl 3β-hydroxychol-5-en-24-oate (12)**. 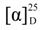 −9.0 (*c* 0.73, CHCl_3_); selected ^1^H NMR (400 MHz CDCl_3_): δ_H_ 5.34 (d, *J* = 5.0 Hz, 1H), 3.66 (s, 3H), 3.50 (m, 1H), 1.00 (s, 3H), 0.92 (d, *J* = 6.5 Hz, 3H), 0.67 (s, 3H); ^13^C NMR (100 MHz CDCl_3_): δ_C_ 175.1, 141.0, 121.8, 71.9, 57.0, 56.0, 51.7, 50.4, 42.4 (2C), 39.9, 37.5, 35.6 (2C), 32.1 (2C), 31.7, 31.3 (2C), 28.3, 24.5, 21.3, 19.7, 18.5, 12.1; HRMS-ESI *m*/*z* 389.3053 [M + H]^+^, C_25_H_41_O_3_ requires 389.3056.

**Methyl 3β-hydroxy-5α-cholan-24-oate (13)**. A solution of methyl 3β-hydroxychol-5-en-24-oate **12** (100 mg, 0.26 mmol) in THF dry/MeOH dry (10 mL/10 mL, v/v) was hydrogenated in the presence of palladium 5% wt on activated carbon (5 mg). The flask was evacuated and flushed first with argon and then with hydrogen. The reaction was stirred at room temperature under H_2_ for 48 h. The catalyst was filtered through Celite, and the recovered filtrate was concentrated under vacuum to give **13** (100 mg, quantitative yield). An analytic sample was obtained by silica gel chromatography eluting with *n*-hexane/ethyl acetate 8:2 and 0.5% of triethylamine. 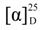 +3.4 (*c* 0.54, CHCl_3_); selected ^1^H NMR (400 MHz, CDCl_3_): δ_H_ 3.64 (s, 3H), 3.56 (m, 1H), 0.89 (d, *J* = 6.0 Hz, 3H), 0.78 (s, 3H), 0.63 (s, 3H).^13^C NMR (100 MHz, CDCl_3_): δ_C_ 175.3, 71.5, 56.7, 56.1, 54.6, 51.8, 45.1, 42.9, 40.3, 38.3, 37.2, 35.7 (2C), 35.6, 32.3, 31.6, 31.3, 31.2, 28.9, 28.4, 24.4, 21.5, 18.5, 12.5, 12.3. HRMS-ESI *m*/*z* 391.3210 [M + H]^+^, C_25_H_43_O_3_ requires 391.3212.

**5α-cholan-3β,24-diol (14)**. To a solution of **13** (50 mg, 0.13 mmol) in dry THF (15 mL) at 0 °C were added, under argon, dry methanol (15 μL, 0.39 mmol) and LiBH_4_ (200 μL, 2M in THF, 0.39 mmol). The resulting mixture was stirred for 2 h at 0 °C. The mixture was quenched by addition of NaOH (1 M, 260 μL) and then allowed to warm to room temperature. Ethyl acetate was added and the separated aqueous phase was extracted with ethyl acetate (3 × 30 mL). The combined organic phases were washed with water, dried (Na_2_SO_4_) and concentrated. Purification by silica gel (*n*-hexane/ethyl acetate 8:2) gave C24 alcohol **14** as a colorless oil (36 mg, 78%).


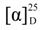
 +21 (*c* 0.08, CH_3_OH); selected ^1^H NMR (400 MHz, CD_3_OD): δ_H_ 3.50 (m, ovl, 2H), 3.50 (m, ovl, 1H), 0.94 (d, *J* = 6.4 Hz, 3H), 0.82 (s, 3H), 0.69 (s, 3H). ^13^C NMR (100 MHz, CDCl_3_): δ_C_ 71.4, 63.7, 56.4, 56.1, 54.3, 44.8, 42.3, 40.0, 38.2 (2C), 36.9 (2C), 35.5, 35.4, 32.1, 32.0, 31.7, 29.3, 28.6, 24.2, 21.2, 18.6, 12.3, 12.0; HRMS-ESI *m*/*z* 363.3260 [M + H]^+^, C_24_H_43_O_2_ requires 362.3263.

**3β-hydroxy-5-cholen-24-oic acid (15)**. A portion of compound **12**(100 mg, 0.26 mmol) was hydrolyzed with lithium hydroxide (18 mg, 0.78 mmol) in a solution of THF/H_2_O 1:1 v/v (5 mL). The resulting solution was then acidified with HCl 6N and extracted with ethyl acetate (3 × 50 mL). The collected organic phases were washed with brine, dried over Na_2_SO_4_ anhydrous and evaporated under reduced pressure to give **15** (77 mg, 80%). An analytic sample was obtained by silica gel chromatography eluting with CH_2_Cl_2_/MeOH 95:5. 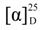 −13.6 (*c* 0.1, CH_3_OH); selected ^1^H NMR (400 MHz, CD_3_OD): δ_H_ 5.35 (d, *J* = 5.0 Hz, 1H), 3.50 (m, 1H), 1.00 (s, 3H), 0.96 (d, *J* = 6.4 Hz, 3H), 0.71 (s, 3H). ^13^C NMR (100 MHz, CD_3_OD): δ_C_ 177.0, 142.5, 122.5, 72.5, 58.2, 57.3, 51.7, 43.5, 43.0, 41.1, 38.6, 36.7, 33.3, 33.0 (2C), 32.3 (2C), 32.0, 29.1, 25.3, 22.2, 19.8, 18.8, 12.3. HRMS-ESI *m*/*z* 375.2895 [M + H]^+^, C_24_H_39_O_3_ requires 375.2899.

**3β-hydroxy-5α-cholan-24-oic acid (16)**. The same procedure of hydrogenation was carried out on a portion of compound **15** (20 mg, 0.05 mmol) to give compound **16** (18 mg, 93%).


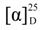
 +4.16 (*c* 1.6, CH_3_OH); selected ^1^H NMR (400 MHz, CDCl_3_): δ_H_ 3.57 (m, 1H), 0.94 (d, *J* = 6.4 Hz, 3H), 0.81 (s, 3H), 0.66 (s, 3H). ^13^C NMR (100 MHz, CD_3_OD): δ_C_ 178.1, 72.0, 57.9, 57.5, 55.8, 46.2, 43.8, 41.4, 38.9, 38.3, 36.9, 36.7, 36.6, 33.3, 32.3, 32.2, 32.0, 30.0, 29.1, 25.3, 22.4, 18.7, 12.8, 12.6. HRMS-ESI *m*/*z* 377.3053 [M + H]^+^, C_24_H_41_O_3_ requires 419.3161.

**Methyl 3α,6α-di-(*tert*-butyldimethylsilyloxy)-5β-cholan-24-oate (18)**. To a solution of methyl hyodeoxycholic-24-oate **17** (1 g, 2.5 mmol) in 30 mL of CH_2_Cl_2_ at 0 °C were added 2,6-lutidine (25 mmol, 2.9 mL) and *tert*-butyldimethylsilyltrifluoromethanesulfonate (7.5 mmol, 1.7 mL). After 2 h stirring at 0 °C, the reaction was quenched by addition of aqueous NaHSO_4_ (1 M, 50 mL). The layers were separated and the aqueous phase was extracted with CH_2_Cl_2_ (3 × 50 mL). The combined organic layers were washed with NaHSO_4_, water, saturated aqueous NaHCO_3_, and brine. Purification by flash chromatography on silica gel, using *n*-hexane/ethyl acetate 95:5 v/v and 0.5% of triethylamine as eluent, gave **18** (1.6 g, quantitative yield) as a clear, colorless oil.


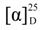
 +2.2 (*c* 0.55, CHCl_3_); selected ^1^H NMR (400 MHz, CDCl_3_): δ_H_ 3.98 (dt, *J* = 3.8, 8.0 Hz, 1H), 3.67 (s, 3H), 3.52 (m, 1H), 0.90 (d, *J* = 6.4 Hz, 3H), 0.88 (s, 9H), 0.87 (s, 9H), 0.86 (s, 3H), 0.62 (s, 3H), 0.04 (s, 6H), 0.02 (s, 6H). ^13^C NMR (100 MHz, CDCl_3_): δ_C_ 175.0, 73.0, 68.6, 56.0, 55.8, 51.4, 49.5, 42.8, 39.9, 39.5 (2C), 35.9 (2C), 35.3 (2C), 34.8, 31.0 (2C), 30.9, 30.8, 29.7, 28.0, 25.9 (3C), 25.5 (3C), 24.2, 23.5, 20.7, 18.2, 12.0, −4.5, −4.7, −4.8, −4.9; HRMS-ESI *m*/*z* 635.4893 [M + H]^+^, C_37_H_71_O_4_Si_2_ requires 635.4891.

**3α,6α-di-(*tert*-butyldimethylsilyloxy)-5β-cholan-24-ol (19)**. Dry methanol (680 μL, 16.8 mmol) and LiBH_4_ (8.4 mL, 2M in THF, 16.8 mmol) were added to a solution of **18** (1.5 g, 2.4 mmol) in dry THF (30 mL) at 0 °C under argon and the resulting mixture was stirred for 1 h at 0 °C. The mixture was quenched by addition of NaOH (1 M, 5 mL). Ethyl acetate was added and the separated aqueous phase was extracted with ethyl acetate (3 × 30 mL). The combined organic phases were washed with water, dried (Na_2_SO_4_) and concentrated. Purification by silica gel (*n*-hexane/ethyl acetate 95:5 and 0.5% of triethylamine) gave alcohol derivative **19** as a colorless oil (1.35 g, 93%).


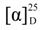
 +6.3 (*c* 0.4, CHCl_3_); selected ^1^H NMR (400 MHz, CDCl_3_): δ_H_ 3.95 (dt, *J* = 3.4, 11.2 Hz, 1H), 3.57 (t, *J* = 6.6 Hz, 2H), 3.50 (m, 1H), 1.92 (m, 1H), 1.83 (m, 1H), 0.90 (d, *J* = 6.6 Hz, 3H), 0.86 (s, 9H), 0.85 (s, 9H), 0.85 (s, 3H), 0.60 (s, 3H), 0.03 (s, 6H), 0.01 (s, 6H). ^13^C NMR (100 MHz, CDCl_3_): δ_C_ 73.0, 68.5, 63.3, 56.0 (2C), 49.5, 42.7, 39.9, 39.5 (2C), 35.8 (2C), 35.5, 35.3, 34.7, 31.7, 30.8 (2C), 29.8, 29.3, 28.1, 25.9 (3C), 25.8 (3C), 24.1, 23.4, 20.7, 18.5, 12.0, −4.5, −4.7, −4.8, −4.9; HRMS-ESI *m*/*z* 607.4940 [M + H]^+^, C_36_H_71_O_3_Si_2_ requires 607.4942.

**Ethyl 3α,6α-di-(*tert*-butyldimethylsilyloxy)-5β-chol-24-en-26-oate (20)**. DMSO (568 μL, 8.0 mmol) was added dropwise over 5 min to a solution of oxalyl chloride (2.0 mL, 4.0 mmol) in dry dichloromethane (10 mL) at −78 °C under argon atmosphere. After 30 min a solution of the alcohol **19** (1.0 g, 1.6 mmol) in dry CH_2_Cl_2_ (5 mL) was added dropwise and the mixture was stirred at –78 °C. After another 30 min, Et_3_N (1.1 mL, 8.0 mmol) was added dropwise to the solution. The reaction after 2 h was quenched by addition of aqueous NaHSO_4_ (1 M, 50 mL). The layers were separated and the aqueous phase was extracted with CH_2_Cl_2_ (3 × 50 mL). The combined organic layers were washed with saturated aqueous NaHSO_4_, saturated aqueous NaHCO_3_ and brine. The organic phase was then dried over Na_2_SO_4_ and concentrated to give the corresponding aldehyde (950 mg) as colorless oil, which was used without any further purification. To a solution of aldehyde (1.57 mmol) in THF dry (10 mL) were added LiOH (41 mg, 1.7 mmol) and TEPA (triethylphosphonoacetate, 342 μL, 1.7 mmol). The reaction mixture was stirred for 1h at room temperature and then quenched with water (10 mL). The mixture was then extracted with ethylacetate (3 × 30 mL), and the organic phase was concentrated *in vacuo*. Flash chromatography (*n*-hexane and 0.5% of triethylamine) afforded compound **20** (1.02 g, 95% over two steps).


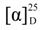
 −0.46 (*c* 0.3, CHCl_3_); selected ^1^H NMR (400 MHz, CDCl_3_): δ_H_ 6.97 (dt, *J* = 5.1, 15.6 Hz, 1H), 5.81 (d, *J* = 15.6 Hz, 1H), 4.19 (q, *J* = 7.5 Hz, 2H), 3.99 (m, 1H), 3.53 (m, 1H), 1.29 (t, *J* = 7.6 Hz, 3H), 0.93 (d, *J* = 6.5 Hz, 3H), 0.90 (s, 9H), 0.90 (s, 3H), 0.89 (s, 9H), 0.64 (s, 3H), 0.06 (s, 6H), 0.04 (s, 6H). ^13^C NMR (100 MHz, CDCl_3_): δ_C_ 166.8, 149.8, 121.0, 72.9, 68.6, 60.0, 56.0 (2C), 49.5, 42.8, 39.9, 39.6, 35.9, 35.8, 35.4 (2C), 34.8, 34.2, 31.0 (3C), 29.8, 28.9, 28.1, 25.9 (3C), 25.8 (3C), 24.2, 23.5, 20.7, 18.4, 14.3, 12.0, −4.5, −4.6, −4.7, −4.8; HRMS-ESI *m*/*z* 675.5200 [M + H]^+^, C_40_H_75_O_4_Si_2_ requires 675.5204.

**Ethyl 3α,6α-di-(*tert*-butyldimethylsilyloxy)-5β-cholan-26-oate (21)**. Compound **20** (1.0 g, 1.48 mmol) and THF dry (25 mL) were mixed and deoxygenated with flowing nitrogen for 5 min. The catalyst Pd 20% wt on carbon (10 mg) was added. The mixture was transferred to a standard PARR apparatus and flushed with nitrogen and then with hydrogen several times. The apparatus was shacked under 50 psi of hydrogen. After 8 h, the reaction was complete. The catalyst was filtered through Celite, and the recovered filtrate was concentrated under vacuum to afford 980 mg of ethyl ester **21** (98%). An analytic sample was obtained by silica gel chromatography, eluting with *n*-hexane/ethyl acetate 95:5. 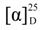 −9.0 (*c* 0.05, CHCl_3_); selected ^1^H NMR (400 MHz, CDCl_3_): δ_H_4.14 (q, *J* = 7.1 Hz, 2H), 4.01 (m, 1H), 3.55 (m, 1H), 2.31 (t, *J* = 7.6 Hz, 2H), 1.28 (t, *J* = 7.1 Hz, 3H), 0.93 (d, *J* = 7.0 Hz, 3H), 0.91 (s, 9H), 0.89 (s, 9H), 0.90 (s, 3H), 0.64 (s, 3H), 0.07 (s, 6H), 0.05 (s, 6H). ^13^C NMR (100 MHz, CD_3_OD): δ_C_ 174.0, 73.0, 68.6, 60.2, 56.2 (2C), 49.6, 42.8, 40.0, 39.8, 39.6, 35.9, 35.6, 35.5, 34.9, 34.4, 31.0 (2C), 30.4, 29.8, 29.6, 28.9, 28.2, 26.0, 25.9 (3C), 25.8 (3C), 24.2, 23.5, 20.8, 18.6, 14.3, 12.0, −4.5, −4.6, −4.7, −4.8. HRMS-ESI *m*/*z* 677.5357 [M + H]^+^, C_40_H_77_O_4_Si_2_ requires 677.5360.

**Ethyl 3α,6α-dihydroxy-5β-cholan-26-oate (22)**. To the ethyl ester **21** (900 mg, 2 mmol), dissolved in ethanol (30 mL), was added 1 mL of HCl 37% v/v and the mixture was stirred for 4 h at room temperature. At the end of reaction, silver carbonate was added to precipitate chloride. Then the reaction mixture was centrifuged and the supernatant was concentrated *in vacuo* to give the desired ethyl ester **22** (900 mg) as a colorless amorphous solid. An analytic sample was obtained by silica gel chromatography eluting with CH_2_Cl_2_/MeOH 95:5.


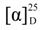
 −3.78 (*c* 0.38, CH_3_OH); selected ^1^H NMR (400 MHz, CDCl_3_): δ_H_ 4.12 (q, *J* = 7.3 Hz, 2H), 4.04 (m, 1H), 3.63 (m, 1H), 2.10 (t, *J* = 7.3 Hz, 2H), 1.25 (t, *J* = 7.3 Hz, 3H), 0.90 (s, 3H), 0.89 (d, *J* = 6.6 Hz, 3H), 0.62 (s, 3H).^13^C NMR (100 MHz, CDCl_3_): δ_C_ 174.2, 71.0, 67.5, 60.2, 56.1, 56.0, 48.4, 42.6, 39.8 (2C), 39.7, 35.7, 35.5, 35.4, 35.3, 34.7, 34.6, 33.9, 29.8, 29.2, 28.1, 25.5, 24.0, 23.5, 20.6, 18.4, 14.3, 12.0. HRMS-ESI *m*/*z* 449.3629 [M + H]^+^, C_28_H_49_O_4_ requires 449.3631.

**Ethyl 3α,6α-ditosyloxy-5β-cholan-26-oate (23)**. To a solution of ethyl ester **22** (850 mg, 1.9 mmol) in dry pyridine (15 mL), a solution of tosylchloride (362 mg, 9.5 mmol) in dry pyridine (15 mL) was added, and the mixture was stirred at room temperature for 4 h. CH_2_Cl_2_ was added and the separated aqueous phase was extracted with CH_2_Cl_2_ (3 × 30 mL). The combined organic phases were washed with water, dried (Na_2_SO_4_) and concentrated. The yellow oily residue was purified through a short column of silica gel (80 g) and eluted with *n*-hexane/ethyl acetate 95:5 and 0.5% of triethylamine. The ditosylate **23** was pure according to the TLC and NMR analyses: 1.3 g (90% over two steps). 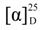 +6.36 (*c* 0.34, CH_3_OH); ^1^H NMR (400 MHz, CDCl_3_): δ_H_ 7.78 (d, *J* = 8.0 Hz, 2H), 7.72 (d, *J* = 8.0 Hz, 2H), 7.35 (d, *J* = 8.0 Hz, 2H), 7.33 (d, *J* = 8.0 Hz, 2H), 4.78 (m, 1H), 4.30 (m, 1H), 4.12 (q, *J* = 7.1 Hz, 2H), 2.28 (t, *J* = 7.5 Hz, 2H), 1.26 (t, *J* = 7.2 Hz, 3H), 0.86 (d, *J* = 6.6 Hz, 3H), 0.80 (s, 3H), 0.60 (s, 3H). ^13^C NMR (100 MHz, CDCl_3_): δ_C_ 173.7, 149.0, 144.2, 135.8, 134.0, 129.3 (2C), 128.6, 127.0, 126.9, 124.9, 123.4, 123.3, 81.4, 79.3, 59.7, 55.4, 55.3, 49.7, 45.8, 42.2, 39.1, 38.9, 35.6, 34.9, 34.8, 34.3, 33.9, 31.6, 27.5, 26.8, 25.9, 25.0, 24.8, 23.4 (2C), 22.3, 21.1, 19.9, 18.0, 13.8, 11.4; HRMS-ESI *m*/*z* 757.3805 [M + H]^+^, C_42_H_61_O_8_S_2_ requires 757.3808.

**Ethyl 3β-hydroxy-5-cholen-26-oate** (**24**). A solution of ethyl 3,6-ditosyloxy-5β-cholan-26-oate **23** (1.0 g, 1.3 mmol) and CH_3_COOK (129 mg, 1.3 mmol) dissolved in water (2 mL) and *N*,*N*′-dimethylformamide (DMF, 14 mL) was refluxed for 4 h. The solution was cooled at room temperature and then ethyl acetate and water were added. The separated aqueous phase was extracted with ethyl acetate (3 × 30 mL). The combined organic phases were washed with water, dried (Na_2_SO_4_) and evaporated to dryness to give 650 mg of mixture, that was subjected to the next step without any purification. This compound was dissolved in 32 mL of mixture CHCl_3_/MeOH (5:3). Then to a solution was added *p*-toluensulfonic acid (*p*TsOH) (500 mg, 2.6 mmol). The mixture was quenched by addition of NaHCO_3_ solution (30 mL) and then concentrated *in vacuo*. Ethyl acetate and water were added and the separated aqueous phase was extracted with ethyl acetate (3 × 50 mL). The combined organic phases were washed with water, dried (Na_2_SO_4_) and concentrated. Purification by silica gel eluting with *n*-hexane/ethyl acetate 7:3 and 0.5% of triethylamine gave the alcohol **24** as a white solid (436 mg, 78% over two steps). 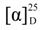 −8.5 (*c* 0.28, CH_3_OH); selected ^1^H NMR (400 MHz, CDCl_3_): δ_H_ 5.31 (d, *J* = 4.2 Hz, 1H), 4.09 (q, *J* = 7.0 Hz, 2H), 3.48 (m, 1H), 2.26 (t, *J* = 7.5 Hz, 2H), 1.23 (t, *J* = 7.0 Hz, 3H), 0.98 (s, 3H), 0.88 (d, *J* = 6.2 Hz, 3H), 0.65 (s, 3H); ^13^C NMR (100 MHz CDCl_3_): δ_C_ 174.0, 140.8, 121.6, 71.7, 60.2, 56.7, 55.9, 50.0, 42.2 (2C), 39.7, 37.2, 36.4, 35.5, 35.4, 34.3, 31.8 (3C), 28.1, 25.5, 25.4, 24.2, 21.0, 19.3, 18.6, 14.2, 11.8; HRMS-ESI *m*/*z* 431.3250 [M + H]^+^, C_28_H_47_O requires 431.3252.

**3β-Hydroxy-5-cholen-26-oic acid** (**25**). Fifty mg of compound **24** (0.12 mmol) was hydrolyzed with lithium hydroxide (14 mg, 0.6 mmol) in a solution of THF/H_2_O 1:1 v/v (4 mL) as described before, to give compound **25** in quantitative yield (48 mg). 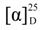 −8.0 (*c* 0.10, CH_3_OH); selected ^1^H NMR (400 MHz, CD_3_OD): δ_H_ 5.33 (d, *J* = 4.3 Hz, 1H), 3.40 (m, 1H), 2.20 (t, *J* = 7.5 Hz, 1H), 1.02 (s, 3H), 0.94 (d, *J* = 6.3 Hz, 3H), 0.71 (s, 3H). ^13^C NMR (100 MHz, CD_3_OD): δ_H_ 177.8, 142.3, 122.5, 72.6, 58.2, 57.6, 51.8, 43.5, 43.0, 41.2, 38.6, 37.7, 37.0, 36.8, 35.0, 33.3, 33.1, 32.3, 29.3, 26.7, 26.5, 25.3, 22.2, 19.9, 19.2, 12.3; HRMS-ESI *m*/*z* 403.3210 [M + H]^+^, C_26_H_49_O_3_ requires 403.3212.

**Methyl 3β-hydroxy-5α-cholen-26-oate** (**26**). An oven-dried 100 mL flask was charged with 10% palladium on carbon (10 mg) and compound **24** (300 mg, 0.7 mmol) and the flask was evacuated and flushed with argon. Absolute methanol (50 mL) and dry THF (50 mL) were added, and the flask was flushed with hydrogen. The reaction was stirred at room temperature under H_2_ for 4 h. The mixture was filtered through Celite, and the recovered filtrate was concentrated to give g of crude product. The residue was subjected to column chromatography on silica gel eluting with *n*-hexane/ethyl acetate 8:2 and 0.5% of triethylamine to give 260 mg of pure **26** (91%). 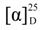 +0.6 (*c* 0.1, CHCl_3_); ^1^H NMR (400 MHz, CDCl_3_): δ_H_ 3.61 (s, 3H), 3.52 (m, 1H), 2.25 (t, *J* = 7.5 Hz, 2H), 0.84 (d, *J* = 6.3 Hz, 3H), 0.75 (s, 3H), 0.59 (s, 3H). ^13^C NMR (100 MHz, CDCl_3_): δ_C_ 174.5, 71.0, 56.3, 56.0, 54.2, 51.3, 44.8, 42.5, 39.9, 38.0, 36.9, 35.4 (2C), 35.3 (2C), 34.0, 31.9, 31.3, 31.0, 28.6, 25.5, 25.3, 24.1, 21.1, 18.5, 12.2, 12.0; HRMS-ESI *m*/*z* 419.3520 [M + H]^+^, C_27_H_47_O_3_ requires 419.3525.

**3β-Hydroxy-5α-cholan-26-oic acid (27)**. Compound **26** (50 mg, 0.12 mmol) was hydrolyzed with a methanol solution of sodium hydroxide (5%, 5 mL) in H_2_O (1 mL) overnight under reflux. The resulting solution was then concentrated under vacuum, diluted with water, acidified with HCl 6N and extracted with ethyl acetate (3 × 50 mL). The collected organic phases were washed with brine, dried over Na_2_SO_4_ anhydrous and evaporated under reduced pressure to give **27** in a quantitative yield (47 mg). An analytic sample was obtained by silica gel chromatography eluting with CH_2_Cl_2_/MeOH 95:5. 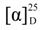 +4.2 (*c* 0.2, CHCl_3_); selected ^1^H NMR (400 MHz, CD_3_OD): δ_H_ 3.50 (m, 1H), 2.28 (t, *J* = 7.6 Hz, 2H), 0.93 (d, *J* = 6.7 Hz, 3H), 0.83 (s, 3H), 0.70 (s, 3H). ^13^C NMR (100 MHz, CD_3_OD): δ_C_ 177.8, 71.9, 57.9, 57.6, 55.8, 46.2, 43.8, 41.4, 38.9, 38.3, 37.0, 36.9, 36.8, 36.6, 35.0, 33.3, 32.2, 30.0, 29.3, 26.7, 26.5, 25.2, 22.4, 19.2, 12.8, 12.6. HRMS-ESI *m*/*z* 405.3367 [M + H]^+^, C_26_H_45_O_3_ requires 405.3369.

**5α-Cholan-3β,26-diol** (**28**). Dry methanol (15 μL, 0.36 mmol) and LiBH_4_ (180 μL, 2 M in THF, 0.36 mmol) were added to a solution of the methyl ester **26** (50 mg, 0.12 mmol) in dry THF (10 mL) at 0 °C under argon and the resulting mixture was stirred for 4 h at 0 °C. The mixture was quenched by addition of NaOH (1 M, 240 μL) and then allowed to warm to room temperature. Ethyl acetate was added and the separated aqueous phase was extracted with ethyl acetate (3 × 15 mL). The combined organic phases were washed with water, dried (Na_2_SO_4_) and concentrated. Purification by silica gel eluting with CH_2_Cl_2_/MeOH (9:1) gave the alcohol **28** as a white solid (36 mg, 78%). 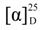 +3.6 (*c* 0.85, CHCl_3_); ^1^H NMR (400 MHz CD_3_OD): δ_H_ 3.53 (t, *J* = 7.2 Hz, 2H), 3.48 (m, 1H), 0.92 (d, *J* = 6.1 Hz, 3H), 0.83 (s, 3H), 0.69 (s, 3H); HRMS-ESI *m*/*z* 391.3573 [M + H]^+^, C_26_H_47_O_2_ requires 391.3576.

### 3.2. Transfection and Luciferase Assays

For PXR mediated transactivation, 5 × 10^4^ HepG2 cells were plated in a 24-well plate and transfected, using Fugene HD transfection reagent (Hoffmann La Roche, Basel, Switzerland), with 75 ng of pSG5-PXR, 75 ng of pSG5-RXR, 125 ng of pCMV-β-galactosidase, and with 250 ng of the reporter vector containing the PXR target gene promoter (CYP3A4 gene promoter) cloned upstream of the luciferase gene (pCYP3A4promoter-TKLuc). At 24 h post-transfection, cells were stimulated with Rifaximin 10 μM (as positive control) and compounds **1**–**8**, **11**–**16** and **24**–**28** 10 μM, or with the combination of 10 μM of Rifaximin and compounds **1**–**8**, **11**–**16** and **24**–**28** 50 μM. In another experimental settings, HepG2 cells were trasfected as described above and primed with increasing doses of **4** (0.1, 1 and 10 μM).

After treatments, cells were lysed in 100 μL Lysis Buffer (25 mM TRIS-phosphate pH 7.8; 2 mM DTT; 10% glycerol; 1% Triton X-100) and 20 μL cellular lysate was assayed for Luciferase activity using the Luciferase Assay System (Promega corporation, Madison, WI, USA). Luminescence was measured using Glomax 20/20 luminometer (Promega corporation, Madison, WI, USA). Luciferase activities were normalized for transfection efficiencies by dividing the Luciferase relative light units (RLU) by β-galactosidase activity (βgal) expressed from cells co-transfected with pCMVβgal. All experiments were performed in triplicate.

### 3.3. Real Time PCR

HepG2 cells were stimulated 18 h with rifaximin (10 μM) or compound **4** (10 μM). Total RNA was extracted using the TRIzol reagent (Invitrogen, Life technology, Carlsband, CA, USA), and reverse-transcribed using random hexamer primers and Super Script-II reverse transcriptase (Invitrogen, Invitrogen, Life technology, Carlsband, CA, USA). mRNA was quantified by Real-Time quantitative PCR on iCycler apparatus (Bio-rad laboratories, Inc., Hercules, CA, USA) using specific primers (hGAPDH: gaaggtgaaggtcggagt and catgggtggaatcatattggaa; hCYP3A4: caagacccctttgtggaaaa and cgaggcgactttctttcatc). For quantitative RT-PCR, 10 ng of template was dissolved in a 20 μL solution containing 200 nM of each primer and 10 μL of KAPA SYBR FAST Universal qPCR Kit (KAPA BIOSYSTEMS, Woburn, MA, USA). All reactions were performed in triplicate, and the thermal cycling conditions were as follows: 3 min at 95 °C, followed by 40 cycles of 95 °C for 15 s, 58 °C for 20 s and 72 °C for 30 s. The relative mRNA expression was calculated accordingly with the *C*_t_ method.

PCR primers were designed using the software PRIMER3 [42] using published sequence data obtained from the NCBI database.

### 3.4. CHiP

HepG2 cells (10^7^) were serum starved for 24 h and then treated for 18 h with rifaximin (10 μM), and with compound **4** (10 μM). After treatment cells were cross-linked with 1% formaldehyde for 10 min at room temperature and then the reaction was stopped by glycine addition, to a final concentration of 125 mM. Cells were washed twice in ice-cold PBS and lysed with 500 μL Swelling Buffer (25 mM Hepes, pH 7.8; 1.5 mM MgCl_2_; 10 mM KCl; 0.1% NP-40; 1 mM DTT) containing protease inhibitors. Cells were centrifuged 2000 rpm for 10 min at +4 °C, re-suspended in Sonication Buffer (50 mM Hepes, pH 7.8; 140 mM NaCl; 1 mM EDTA; 1% Triton X-100; 0.1% SDS) plus protease inhibitors and then sonicated four times for 30″ using Bandelin SONOPULS ultrasonic homogenizers (cycle 8, power 70%). Fifty μL of each supernatant (Input DNA) were reverse-cross-linked by the addition of 150 μL Elution Buffer (1% SDS; 0.1 M NaHCO_3_) and 12 μL NaCl 5 M and by heating the mixture to 65 °C overnight. DNA was recovered from Input by proteinase K treatment at 65 °C for 4 h, followed by phenol/chloroform (1:1) extraction, ethanol precipitation and dissolving in 50 μL TE1x. Thus, 150 μL of Input DNA was diluted in 850 μL of Sonication Buffer containing protease inhibitors and then 20 μL of Sonication Buffer equilibrated Protein A Sepharose (Invitrogen, Life technology, Carlsband, CA, USA)/Salmon Sperm DNA (Invitrogen, Life technology, Carlsband, CA, USA)/1% BSA (PAS/SS/BSA) were added to each sample. After mixing at +4 °C for 1 h, mixtures were centrifuged 2000 rpm for 5 min to obtain supernatants, that were subsequently immunoprecipitated overnight at +4 °C with specific antibodies: anti-SRC1 (sc-32789X, Santa Cruz Biotechnology, Inc., Dallas, TX, USA) or anti-IgG (SA1-36098, Pierce, Thermo Fischer Scientific, Inc., Rockford, IL, USA). Then 40 μL PAS/SS/BSA were added to each mixture, which was incubated at +4 °C for 2 h and then centrifuged 13000 rpm for 1 min. Immunoprecipitates were washed twice with Low Salt Buffer (0.1% SDS; 1% Triton X-100; 2 mM EDTA, pH 8.0; 20 mM Tris–HCl, pH 8.0; 150 mM NaCl), twice with High Salt Buffer (0.1% SDS; 1% Triton X-100; 2 mM EDTA, pH 8.0; 20 mM Tris-HCl, pH 8.0; 500 mM NaCl) and finally once in TE 1× (10 mM Tris-HCl, pH 8.0; 1 mM EDTA, pH 8.0). DNA was eluted by addition of 250 μL Elution Buffer and the cross-linking reactions were reversed by heating the mixture to 65 °C overnight. The DNA was recovered from immunoprecipitated material by proteinase K treatment at 65 °C for 4 h followed by phenol/chloroform (1:1) extraction, ethanol precipitation and dissolving in 20 μL TE1x. Two microliters chromatin was used for quantitative real-time PCR for the amplification of the CYP3A4 promoter. The sequences of primers used for the amplification of the proximal promoter region of the CyP3A4 gene were: ATGCCAATGGCTCCACTTGAG and CTGGAGCTGCAGCCAGTAGCAG. Raw data analysis was performed as follows: Δ*C*_t_ was calculated *vs.* the input DNA concentration; ΔΔ*C*_t_ was *vs.* unstimulated cells immunoprecipitated with the anti-IgG antibody (experimental condition set as 1.0); the relative expression was calculated as 2^−(ΔΔ*C*t)^.

### 3.5. Statistical Analysis

All values are expressed as the mean ± SD. Comparisons of more than two groups were made with a one-way analysis of variance with *post-hoc* Tukey tests. Differences were considered statistically significant if *p* was <0.05.

### 3.6. Computational Methods

#### 3.6.1. Ligand and Protein Preparation

The tridimensional structures of compound **4** was generated with the Maestro Build Panel [43] and then submitted to Polak-Ribiere conjugate gradient minimization (0.0005 kJ/(Å mol) convergence) using MacroModel (version 9.9) [44]. The crystal structure of the PXR-LBD (PDB code 3hvl) was prepared using the “Protein Preparation Wizard” panel of the Schrödinger 2012 molecular modeling package [36]. Thus, the bond orders and disulfide bonds were assigned, all the hydrogen atoms were added, and all the water molecules were deleted. An optimization of the hydrogen-bonding network was performed using the “H-bond assignment” tool. Finally, using the “impref utility”, the positions of the hydrogen atoms were optimized by keeping all the heavy atoms in place.

#### 3.6.2. Docking Calculations

Docking studies were carried out with Glide v. 5.8 (Schrödinger) [45]. Glide is a grid-based ligand docking with energetics approach and searches for favorable interactions between ligands and receptors. The shape and properties of the receptor are represented on a grid by different sets of fields that provide progressively more accurate scoring of the ligand pose. These fields are generated as preprocessing steps in the calculation and hence need to be computed only once for each receptor. For the grid generation, a box centered on the PXR ligand binding cavity was created. This box gives a more precise measure of the effective size of the search space. However, ligands can move outside this box during grid minimization. The Cartesian coordinates of the outer box, X, Y, and Z length were set to 20 Å. The conformational space of the ligand is defined by Glide by several lowest-energy poses that are subjected to a Monte Carlo procedure that examines nearby torsional minima. This procedure is needed in some cases to properly orient peripheral groups and occasionally alters internal torsion angles. The default value (1.00) for the van der Waals radii scaling factor was chosen, which means no scaling for the nonpolar atoms was performed. In the present study, the extra precision (XP) mode of GlideScore function was used to score the obtained binding poses. The force field used for the docking was the OPLS-2005 [46].

All of the pictures were rendered with PyMOL [47].

## 4. Conclusions

The peculiar property of nuclear receptors is the ability to directly interact with genomic DNA and control the expression of specific genes. As a consequence, nuclear receptors play key roles in both embryonic development and adult homeostasis. In this study we report the pharmacological evaluation of five hydroxysteroids, isolated from *Sinularia kavarattiensis*, and several newly synthesized hydroxysteroid derivatives, endowed with different side chains. The subsequent biochemical characterization of these analogues allowed us to identify (24*S*)-ergosta-5-en-3β-ol, compound **4**, as a new potent PXR agonist. In particular, this ligand was tested through transactivation assays using HepG2 cells transiently transfected with a PXR vector. Prompted by the promising biological results we decided to investigate the binding mechanism of **4** to the PXR through docking simulations. Our results elucidate the most relevant ligand/receptor interactions, allowing to detect the ligand structural requirements for PXR agonism. Compound **4**, also known as dihydrobrassicasterol, is together with its 24*R* epimer, campesterol, β-Sitosterol, and stigmasterol, one of the main components of the phytosterol mixtures of vegetables and vegetable products, such as vegetable oil, olive oil, fruit and nuts [48]. Plant sterols are proven to exert health benefits via the lowering of low density lipoprotein cholesterol concentration [49]. Our study reports the first example of a plant sterol acting as a potent PXR agonist. Although variability in plasma concentration of plant sterols is large across and within different population groups, concentrations of campesterol and dehydrobrassicasterol also referred to as “campesterol fraction” were reported to range from 6.9 to 27.9 μM [50], values that make the calculated EC_50_ in the *in vitro* transactivation assays of physiological relevance, thus opening new opportunities for further investigations.
